# Actions of CSF2 and DKK1 on bovine embryo development and pregnancy outcomes are affected by composition of embryo culture medium

**DOI:** 10.1038/s41598-022-11447-7

**Published:** 2022-05-07

**Authors:** Thiago F. Amaral, Joao Gabriel Viana de Grazia, Luany Alves Galvao Martinhao, Felipe De Col, Luiz Gustavo B. Siqueira, Joao Henrique M. Viana, Peter J. Hansen

**Affiliations:** 1 Department of Animal Sciences, D.H. Barron Reproductive and Perinatal Research Program, and Genetics Institute, University of Florida, Gainesville, FL 32611-0910 USA; 2 FIVX Apoyar Biotech LTDA, Juiz de Fora, MG Brazil; 3 Embrapa Gado de Leite, Juiz de Fora, MG 36038-330 Brazil; 4 Embrapa Recursos Genéticos E Biotecnologia, Brasilia, DF 70770-901 Brazil; 5 Present Address: Zoetis, Kalamazoo, MI 49007 USA; 6 Present Address: Biological Science Institute, University of Brasilia, Brasilia, DF Brazil

**Keywords:** Reproductive biology, Embryology

## Abstract

Procedures for in vitro embryo production in cattle have not been optimized. In the current experiment, we utilized a 3 × 3 factorial design to test whether the proportion of embryos becoming blastocysts in culture and the pregnancy rate after embryo transfer are affected by type of serum in the medium [no serum; 3% (v/v) KnockOut Serum Replacement (SR); 3% (v/v) fetal bovine serum (FBS)] and addition of specific embryokines [vehicle; 10 ng/mL colony stimulating factor 2 (CSF2); 100 ng/mL dickkopf related protein 1 (DKK1)] at day 5 of culture. Embryos were produced using abattoir-derived ovaries and Y-sorted semen from two Angus sires. The percent of putative zygotes and cleaved embryos becoming blastocysts was improved by SR and FBS. Pregnancy rate at day 30 was determined for 1426 Nelore recipients and calving rate for 266 recipients. In the absence of CSF2 or DKK1, pregnancy rates were lower for embryos cultured with SR or FBS. CSF2 and DKK1 reduced pregnancy rate for embryos cultured without serum but had no detrimental effect in the SR or FBS groups. Indeed, CSF2 blocked the negative effect of FBS on pregnancy rate. Data on birth weights were available for 67 bull calves. There were no effects of treatment. The sire used to produce embryos had significant and large effects on development to the blastocyst stage, pregnancy rate at day 30, calving rate and pregnancy loss between day 30 and calving. Results indicate that (1) SR and FBS can improve embryonic development in vitro while also compromising competence of embryos to survive after transfer, (2) actions of CSF2 and DKK1 depend upon other characteristics of the embryo production system, and (3) sire can have a large effect on embryonic development before and after transfer.

## Introduction

In vitro production of embryos has become the predominant method for producing bovine embryos for transfer into recipient females^1^. Nonetheless, procedures for producing embryos in vitro are not optimized. The proportion of embryos developing to the blastocyst stage is usually less than 40% while as many as 50–80% of embryos developing in vivo become blastocysts^2^. Moreover, pregnancy rates after embryo transfer are often less than what is achieved using embryos produced in vivo^3–5^.

One strategy for improving systems for in vitro production of embryos is to provide additives to embryo culture medium that stimulate development or improve competence of embryos to establish pregnancy. Inclusion of fetal bovine serum (FBS) to embryo culture medium can increase the percent of embryos becoming a blastocyst^6–10^ but concerns have been expressed that serum, which is an undefined fluid, contains molecules that promote abnormal development resulting in large offspring syndrome^3,11,12^. There is experimental evidence for this idea in sheep^13,14^. There is also one report that competence of bovine embryos to establish pregnancy after embryo transfer was reduced by FBS^6^. A substitute for serum called KnockOut Serum Replacement (SR) has been reported to increase blastocyst yields to a similar degree as serum^9^. Although free of serum, the composition is proprietary and could be undefined. Another complex solution, oviductal fluid, increased the percent of bovine embryos becoming blastocysts when added at low concentrations (up to 1.25%) but was deleterious at concentrations of 5% or above^15^.

An alternative approach to improve embryo yield and quality is to add specific embryokines to the culture medium. An embryokine is defined as a regulatory molecule produced by the reproductive tract that modulates embryonic development^16^. Several molecules produced by the reproductive tract can increase development to the blastocyst stage in vitro^17,18^. A smaller number of embryokines have been reported to increase embryo pregnancy rate after transfer to recipients. Besides insulin-like growth factor 1, which increases pregnancy rate during heat stress^19^, two other embryokines reported to increase embryo competence to establish pregnancy rate when added to embryo culture medium from day 5 to 7 of development are the WNT antagonist dickkopf WNT signaling pathway inhibitor 1 (DKK1) and the cytokine colony stimulating factor 2 (CSF2)^20,21^.

DKK1 is produced by the bovine endometrium^22^. Treatment of embryos with DKK1 decreased the canonical WNT signaling molecule β-catenin (CTNNB1)^23^ and increased the proportion of cells in the blastocyst that were trophectoderm^21^. Moreover, treatment with DKK1 from day 5 to 7 increased trophoblast length and interferon-τ accumulation in the uterus at day 15 after ovulation^24^. Colony stimulating factor 2, also called granulocyte–macrophage colony stimulating factor, is also produced by the endometrium of the cow during the first few days after ovulation^22^. Treatment of bovine embryos with CSF2 beginning at day 5 of development has been reported to increase the percent of embryos becoming blastocysts^20,25,26^, block effects of heat shock on apoptosis^27^ and induce sex-dependent changes in both gene expression in the blastocyst^28^ and subsequent elongation of the embryo at day 15 of gestation^29^.

There are two reports with Holstein embryos produced using X-sorted semen and transferred into lactating Holstein cows that CSF2 can increase competence of embryos to establish pregnancy^20,21^. In contrast, there was no effect of CSF2 in another experiment with Holstein embryos produced with X-sorted semen^20^ or in an experiment with Brahman embryos produced with conventional semen^10^. Neither CSF2 nor DKK1 improved pregnancy rate of embryos produced in the presence of serum and transferred into beef cattle recipients^30^. These latter results suggest that the action of embryokines like DKK1 and CSF2 depend upon other characteristics of the embryo and recipient, including possibly the sex or breed of embryo, breed of recipient, and presence of serum in the culture medium.

Another important contributor to the formation and eventual fate of the embryo is the sire. The specific bull that provided semen for in vitro fertilization can affect the proportion of embryos that develop to the blastocyst stage in culture^31–34^. There is also one report with a limited number of embryos that sire can affect post-transfer survival^35^. A better understanding of the impact of sire on embryo survival could lead to improvements in embryo transfer programs.

The current experiment was focused on exploring modifications of embryo culture medium based on SR or FBS and addition of either CSF2 or DKK1. Embryos were produced using Y-sorted semen so that more than 90% of embryos were male. Recipients were Nelore beef cows. The first hypothesis tested was that both SR and FBS would increase the percent of in vitro produced embryos that became blastocysts without having a negative effect on blastocyst capacity to establish pregnancy after transfer. The second hypothesis was that CSF2 and DKK1 would increase blastocyst competence to establish pregnancy and that CSF2 would also increase blastocyst yield. The third hypothesis was that effects of embryokines would be dependent on the presence of serum and that the biologically-active molecules in SR and FBS would negate any additional benefits of addition of CSF2 or DKK1. Embryos were produced using two sires; an additional objective was to test the impact of sire on embryonic development in culture and embryonic survival after transfer.

## Methods

All procedures involving cows were approved by the Animal Care and Use Committee of the University of Florida and all methods were performed in accordance with the relevant guidelines and regulations. The study is reported in accordance with ARRIVE guidelines (www.arriveguidelines.org).

### Oocyte collection, maturation and fertilization

The experiment was conducted using cumulus–oocyte-complexes (COC) recovered from aspiration of 2 to 8 mm visible follicles on the cortex of ovaries from Nelore cows (*Bos indicus*) collected at slaughter from the Alvorada abattoir (Alta Foresta, MT, Brazil) located 8 km from the laboratory. The COC were collected during the summer from September 2019 to February 2020. Ovaries were transported to the laboratory suspended in sterile saline at room temperature. Once in the laboratory, ovaries were rinsed in saline at 36 °C, dried with a paper towel and subjected to manual aspiration using a 10 mL syringe and 18 G needle. The aspirate was transferred to a 50 mL tube and COC allowed to settle to the bottom of the tube. After formation of a visual pellet, the supernatant was aspirated with a 5 mL glass pipette, the pellet was resuspended with 20 mL of saline at 36 °C and the procedure for collection and washing of the pellet repeated three times. Then the contents were placed in 100 mm × 20 mm search plates (cell culture treated, nonpyrogenic polystyrene, Corning, NY, USA). The COC with at least three layers of cumulus cells and homogeneous oocyte cytoplasm were selected for maturation.

Selected COC were washed three times in maturation medium and transferred in groups of 30 into cryotubes (5 mL polystyrene round-bottom tube, Falcon, Brookings, SD, USA) with 400 µL maturation medium overlaid with 200 µL mineral oil (Irvine Scientific, Santa Ana, CA, USA). The maturation medium was Tissue Culture Medium 199 with Earle’s salts (Sigma-Aldrich, St. Louis, MO, USA) supplemented with 22 µg/mL pyruvate (Sigma-Aldrich); 50 µg/mL amikacin (Sigma-Aldrich); 10% (v/v) FBS (Sigma-Aldrich); 0.5 µg/mL follicle stimulating hormone (Folltropin; Vetoquinol, Indianópolis—São Paulo, SP, Brazil); 50 µg/mL human chorionic gonadotropin (Chorulon, MSD, São Paulo, SP, Brazil); 1 µg/mL estradiol (Sigma-Aldrich); 6.25 µg/mL insulin (Sigma-Aldrich) and 1 µL/mL β-mercaptoethanol (Sigma-Aldrich).

Maturation was allowed to proceed for 22 h at 38.5 °C and 5.5% CO_2_. Matured COC were washed twice in in vitro fertilization (IVF) medium and transferred into 50 µL drops of medium overlaid with mineral oil and containing 2 × 10^6^/mL Y-sorted semen from one of two different Angus sires. The semen was purified using PureSperm^®^ (NidaCon International AB, Mölndal, Sweeden) according to manufacturer’s guidelines. Each fertilization drop contained 30 COC and sperm; co-incubation was performed for 18 h at 38.5 °C and 5.5% CO_2_. The fertilization medium was Tyrode’s albumin-lactate-pyruvate supplemented with 50 µg/mL amikacin; 6 mg/mL essentially fatty-acid free BSA; 40 µL/mL of a solution of 2 µM penicillamine; 1 µM hypotaurine and 0.25 µM epinephrine and 10 µg/mL heparin (all from Sigma-Aldrich).

### Embryo culture

Composition of the embryo culture medium used as a base to prepare treatments is provided in Supplementary Table S1. Putative zygotes were assigned randomly to one of three culture media. The no-serum medium was prepared by supplementing 10.3 mL of medium with 50 μL of 50 µg/mL amikacin and 0.03 g essentially fatty-acid free BSA. The SR medium was prepared by supplementing 10.0 mL of medium with 300 μL Gibco KnockOut Serum Replacement (Thermo Fisher Scientific, Waltham, MA, US), 50 μL of 50 µg/mL amikacin and 0.03 g essentially fatty-acid free BSA. The FBS medium was prepared by supplementing 10.0 mL of medium with 300 μL FBS (Thermo Fisher Scientific), 50 μL of 50 µg/mL amikacin and 0.03 g essentially fatty-acid free BSA. Putative zygotes (i.e., oocytes exposed to sperm) were washed and manually denuded by subsequent pipetting in drops of fertilization medium. Zygotes were then assigned randomly to culture medium, washed three times in the culture medium to which they were assigned, and then placed in groups of up to 30 in 60 µL drops of culture medium overlaid with mineral oil (Irvine). Embryos were cultured at 38.5 °C in a humidified atmosphere of 5.5% O_2_ and 5% CO_2_. At day 3 after initiation of fertilization, 50% of the medium was replaced with fresh medium and cleavage was assessed. At day 5, 6 µL of medium was removed and replaced with either vehicle (1000 ng/mL essentially fatty-acid free BSA in Dulbecco’s phosphate buffered saline), 100 ng/mL recombinant bovine CSF2 (Kingfisher Biotech; St. Paul, MN, USA), or 1000 ng/mL recombinant human DKK1 in vehicle (R&D Systems, Minneapolis, MN, USA). Thus, final concentrations after dilution with the medium in each drop were 10 ng/mL CSF2 or 100 ng/mL DKK1. These concentrations have been reported to affect several aspects of embryo physiology and development including competence of embryos to establish pregnancy after transfer into recipients^10,20,21,23–29^. Embryos were cultured until day 7 after initiation of fertilization at 38.5 °C and an atmosphere of 5.5% O_2_ and 5% CO_2_. Blastocyst stage of development and quality were then recorded and scored according to the International Embryo Technology Society (IETS) guidelines^36^. Grade 1 blastocysts were loaded individually into 0.25 mL straws with embryo culture medium supplemented with 5% (v/v) fetal bovine serum, 0.8 mg/mL HEPES, 3 mg/mL essentially fatty-acid free BSA, and 0.25 µg/mL amikacin and shipped to the farm in a portable incubator at 37 °C (WTA, Cravinhos, SP, Brazil) for transfer into recipients.

### Embryo transfer and evaluation of pregnancy outcomes

Recipients were suckled Nelore primiparous and multiparous cows maintained on 7 separate farms. Within each farm, recipients were assigned at random to one of the nine treatments. Estrous cycles of recipients were synchronized as follows. On day-8, females were administered an intravaginal progesterone device (1.9 g, CIDR^®^, Zoetis, São Paulo, SP, Brazil) and 2 mg estradiol benzoate, i.m. (Gonadiol^®^, Zoetis, Brazil). On Day-2, the CIDR inserts were removed and cows were administered, i.m., 12.5 mg PGF_2α_ (Lutalyse^®^, Zoetis), equine chorionic gonadotropin (eCG; Novormon®, Zoetis; 300 IU) and 0.6 mg estradiol cypionate (ECP^®^; Zoetis) to induce ovulation (day 0). On day 7 after expected ovulation, the ovaries of all cows were examined by transrectal ultrasonography using an 5–8 MHz linear-array probe (SonoScape E5, Shenzhen, China) to determine the presence or absence of a corpus luteum. Only cows with a visible corpus luteum were selected as recipients.

On day 7, a single embryo was transferred to the uterine horn ipsilateral to the ovary bearing a corpus luteum (n = 1565). Pregnancy diagnosis by ultrasound was performed at 30 (n = 1436 recipients) days of gestation. All but 74 transfers and all pregnancy diagnoses were performed by a single veterinarian. Data on calving (n = 266 recipients from one farm) and data on birth weight of male calves (n = 23 from sire 1 and n = 44 from sire 2; all from one farm) were available for a subset of animals. Weights were measured within 96 h after birth. Regression analysis was used to adjust weights to predicted weight at 0–24 h after birth.

### Statistical analysis

Data were analyzed using the Statistical Analysis System version 9.4 (SAS Institute, Cary, NC, USA). The statistical model included effects of medium (no serum, SR, and FBS), embryokine (vehicle, CSF2, and DKK1), sire, farm and interactions between medium, embryokine and sire. Orthogonal contrasts were used to partition degrees of freedom for effects of medium into variance due to no serum vs other treatments and SR vs FBS. Similarly, contrasts were used to partition embryokine degrees of freedom into variance due to vehicle vs CSF2 and vehicle vs DKK1. Contrasts were also used to partition variance due to the medium × embryokine interaction based on the contrasts described above.

Binary response variables were analyzed by logistic regression models fitted to a binomial distribution using the GLIMMIX procedure. For development data, binary response variables were the percent of presumptive zygotes that cleaved after fertilization, presumptive zygotes becoming blastocysts at day 6 and day 7, and cleaved embryos becoming blastocysts. Pregnancy outcomes included pregnancy rate at day 30, calving rate, and pregnancy loss between day 30 and calving. Statistical models were as described above. In addition, interactions of sire with other treatments affecting pregnancy rate at day 30 was also examined in a subset of data from the 5 of 7 farms in which embryos from both sires were used. Chi-square analysis was also used to analyze effects on pregnancy loss.

Continuous variables analyzed were gestation length and adjusted birth weight (male calves only). Analysis was by analysis of variance using the GLM procedure of SAS. The model for gestation length included fixed effects of medium, embryokine, the interaction and sire. Adjusted birth weight was analyzed similarly with and without gestation length as a covariate.

## Results

### Embryo development

**Figure 1:**
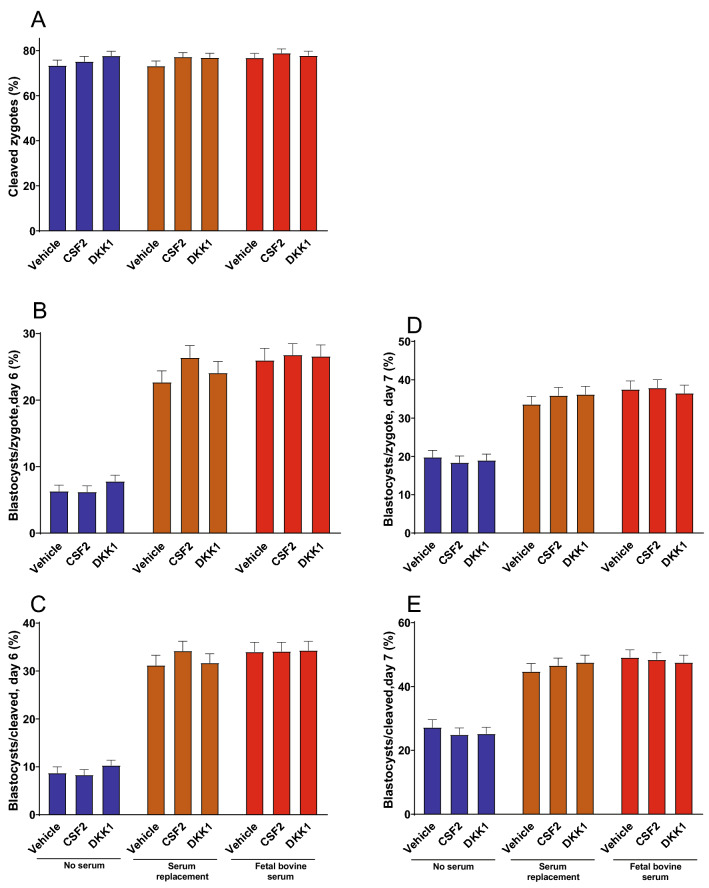
Effects of medium and embryokine treatments on development of bovine embryos. Cleavage was measured at day 3 (**A**) and development to the blastocyst stage at day 6 (**B**,**C**) and 7 (**D**,**E**). Media treatments were no serum, serum replacement (SR) and fetal bovine serum (FBS). Embryokine treatments were added at day 5 and were vehicle, colony stimulating factor 2 (CSF2) and dickopf WNT signaling pathway inhibitor 1 (DKK1). Shown are least-squares means ± SEM. Cleavage rate was affected by medium (P = 0.029) and embryokine (P = 0.008). Using contrasts, cleavage was higher (P = 0.027) for FBS than SR. Cleavage was also lower (P = 0.012) for vehicle than for CSF2 or DKK1. The proportion of embryos that became blastocysts at day 6 (**B**,**C**) or day 7 (**D**,**E**), whether expressed as the percent of putative zygotes (**B**,**D**) or cleaved embryos (**C**,**E**), was affected by medium (P < 0.0001). Further analysis using orthogonal contrasts indicated that more embryos became blastocysts in media with SR or FBS than for no serum (P < 0.0001).The total number of putative zygotes/treatment were 945 for no serum—vehicle, 1165 for no serum—CSF2, 1216 for no serum DKK1, 1288 for SR—vehicle, 1416 for SR and CSF2, 1526 for SR and DKK1, 1288 for FBS and vehicle, 1551 for FBS and CSF2, and 1593 for FBS and DKK1.

Effects of medium and embryokine treatments on cleavage and development are shown in Fig. 1. Cleavage rate was affected by medium (P = 0.029) and embryokine (P = 0.008) but not the interaction (Fig. 1A). Further analysis using contrasts indicated that cleavage was higher (P = 0.027) for FBS (77.9 ± 1.7%) than SR (75.8 ± 1.8%) and that no serum (75.5 ± 1.9%) was not different (P = 0.144) than SR + FBS. Cleavage was also slightly lower (P = 0.012) for vehicle (74.5 ± 1.9%) than for CSF2 (77.1 ± 1.7%; P = 0.012) or DKK1 (77.5 ± 1.7%; P = 0.004). However, these differences were due to chance because embryokine treatment was applied after cleavage was measured.

The proportion of embryos that became blastocysts at day 6 (Fig. 1B,C) or day 7 (Fig. 1D,E), whether expressed as the percent of putative zygotes (Fig. 1B,D) or cleaved embryos (Fig. 1C,E), was affected by medium (P < 0.0001) but not by embryokine or the medium × embryokine interaction. Further analysis using orthogonal contrasts indicated that more embryos became blastocysts in media with SR or FBS than for no serum (P < 0.0001).

### Pregnancy outcomes at day 30

Data on the percent of cows receiving an embryo that were pregnant at day 30 of gestation is shown in Table 1. Odds-ratios were calculated with the no serum—vehicle group as the reference. Based on non-overlapping confidence intervals with odds ratio of 1, pregnancy rate was higher for cows receiving no serum-vehicle embryos than for any other treatment except for cows receiving FBS + CSF2 embryos. Statistical analysis was also performed using a 3 × 3 factorial model with main effects of medium and embryokine. There was no overall effect of medium (P = 0.125) or embryokine (P = 0.427) but separation of effects into individual degree of freedom contrasts indicated that pregnancy rate was lower (P = 0.056) for SR (198/548; 36.13%) than FBS (266/620; 42.9%). The interaction of medium and embryokine was significant (P = 0.042). Furthermore, separation of effects into individual degree of freedom contrasts indicated that differences between no serum vs (SR + FBS) were different for CSF2 (P = 0.004) and DKK1 (P = 0.015) as compared to vehicle. In particular, pregnancy rates were lower for SR and FBS than for no serum when no embryokine was present. Addition of either CSF2 or DKK1 lowered pregnancy rate for cows receiving embryos cultured in no serum but there was no additional reduction in pregnancy rate due to CSF2 or DKK1 for cows receiving embryos cultured in SR or FBS. In fact, CSF2 increased pregnancy rate for embryos cultured in serum (96/196; 49.0% for CSF2 vs 54/138; 39.1% for vehicle).

**Table 1:** Effect of culture medium and embryokine treatment of embryos on pregnancy outcome at day 30 of gestation.

Medium	Embryokine	Pregnant, fraction and percent^a^	Odds ratio^b^	Odds ratio for embryokine within each medium^b^	Odds ratio for medium within vehicle^b^	Odds ratio for medium within CSF2^b^	Odds ratio for medium within DKK1^b^
No serum	Vehicle	45/81 (55.6%)	1	1	1		
No serum	CSF2	35/94 (37.2%)	0.475 (0.256 0.881)	0.472 (0.252 0.887)		1	
No serum	DKK1	35/93 (37.6%)	0.470 (0.253 0.873)	0.468 (0.249 0.879)			1
Knockout serum replacement	Vehicle	58/165 (35.2%)	0.450 (0.259 0.781)	1	0.442 (0.252 0.776)		
Knockout serum replacement	CSF2	67/182 (36.8%)	0.469 (0.271 0.810)	1.160 (0.738 1.823)		1.097 (0.637 1.890)	
Knockout serum replacement	DKK1	73/201 (36.3%)	0.482 (0.283 0.821)	1.073 (0.691 1.667)			1.010 (0.594 1.718)
Fetal bovine serum	Vehicle	54/138 (39.1%)	0.524 (0.298 0.924)	1	0.505 (0.284 0.896)		
Fetal bovine serum	CSF2	96/196 (49.0%)	0.758 (0.447 1.286)	1.460 (0.925 2.305)		1.582 (0.937 2.671)	
Fetal bovine serum	DKK1	116/286 (40.6%)	0.599 (0.360 0.994)	1.108 (0.714 1.720)			1.238 (0.743 2.061)

Abbreviations are as follow: *BSA* bovine serum albumin, *CSF2* colony stimulating factor 2, *DKK1* dickopf WNT signaling pathway inhibitor-1.

^a^Pregnancy rate was affected by the interaction between medium and embryokine (P = 0.042) and by the contrast comparing serum replacement (SR) vs fetal bovine serum (FBS) (P = 0.056) and the interaction between effects of BSA vs SR + FBS and CSF2 (P = 0.004) and DKK1 (P = 0.015).

^b^Odds ratios were calculated after adjustment for farm, sire and the interaction of sire with treatment.

Examination of the odds ratios calculated relative to vehicle for each medium resulted in a similar conclusion as described in the previous paragraph (Table 1). Based on non-overlapping confidence intervals, both CSF2 and DKK1 reduced pregnancy rate compared to vehicle in the no serum group but not in the SR or FBS groups. Analysis of odds ratios also indicated that both SR or FBS reduced pregnancy rate in the vehicle group but not in the CSF2 or DKK1 groups.

### Calving outcomes

Data on calving outcomes were available for a total of 266 recipients from one farm, of which 72 calved. There was no overall effect of medium (P = 0.647), embryokine (P = 0.983) or the interaction (P = 0.300) on pregnancy rate at day 30 although, numerically, results paralleled the larger data set presented in Table 2. There was a tendency for an interaction between no serum vs (SR + FBS) and vehicle vs DKK1 (P = 0.084). Calving rate was higher for cows receiving embryos cultured without serum than for cows receiving embryos cultured with SR or FBS (orthogonal contrast, P = 0.027). There were interactions between the effects of media [no serum vs (SR + FBS)] and the effects of vehicle vs CSF2 (P = 0.050) and DKK1 (P = 0.051). The interactions resulted because CSF2 and DKK1 reduced the calving rate in the absence of serum but increased calving rate in the presence of serum replacement or serum.

**Table 2:** Effect of culture medium and embryokine treatment of embryos on pregnancy outcome at day 30 of gestation and calving for one farm in which data at calving were recorded.

Medium	Embryokine	Pregnant at day 30, fraction and percent^a^	Calving rate, fraction and percent^b^	Pregnancy loss, day 30 to calving, fraction and percent^c^
No serum	Vehicle	10/18 (55.6%)	8/18 (44.4%)	2/10 (20.0%)
No serum	CSF2	4/13 (30.8%)	2/13 (15.4%)	2/4 (50.0%)
No serum	DKK1	5/16 (31.3%)	5/16 (31.3%)	0/5 (0.0%)
Knockout serum replacer	Vehicle	8/25 (32.0%)	2/25 (8.0%)	6/8 (75.0%)
Knockout serum replacer	CSF2	17/41 (41.5%)	7/41 (17.1%)	10/17 (58.8%)
Knockout serum replacer	DKK1	9/25 (36.0%)	7/25 (28.0%)	2/9 (22.2%)
Fetal bovine serum	Vehicle	13/40 (32.5%)	7/40 (17.5%)	6/13 (46.2%)
Fetal bovine serum	CSF2	17/39 (43.6%)	14/39 (35.9%)	3/17 (17.7%)
Fetal bovine serum	DKK1	26/49 (53.1%)	20/49 (40.8%)	6/26 (23.1%)

Abbreviations are as follow: *BSA* bovine serum albumin, *CSF2* colony stimulating factor 2, *DKK1* dickopf WNT signaling pathway inhibitor- 1.

^a^There was an interaction between no serum vs (SR + FBS) and vehicle vs DKK1 (P = 0.084).

^b^Calving rate was affected by medium (P = 0.079). Analysis of contrasts indicated that serum replacement (SR) differed from fetal bovine serum (FBS) (P = 0.027), and there were interactions between no serum vs (SR + FBS) and vehicle vs CSF2 (P = 0.050) and no serum vs (SR + FBS) and vehicle vs DKK1 (P = 0.051).

^c^There were no effects of medium, embryokine or the interaction (P ≥ 0.10).

Using Proc GLIMMIX, there were no significant effects on pregnancy loss between day 30 of gestation and calving, which included 37 of the 109 pregnancies at day 30 (33.9%). Analysis by chi-square, however, was indicative of an effect of medium on pregnancy loss (P = 0.017), with pregnancy loss lower in the no serum (4/19; 21.1%) and FBS groups (15/56; 26.8%) than for the SR group (18/34; 52.9%).

**Figure 2:**
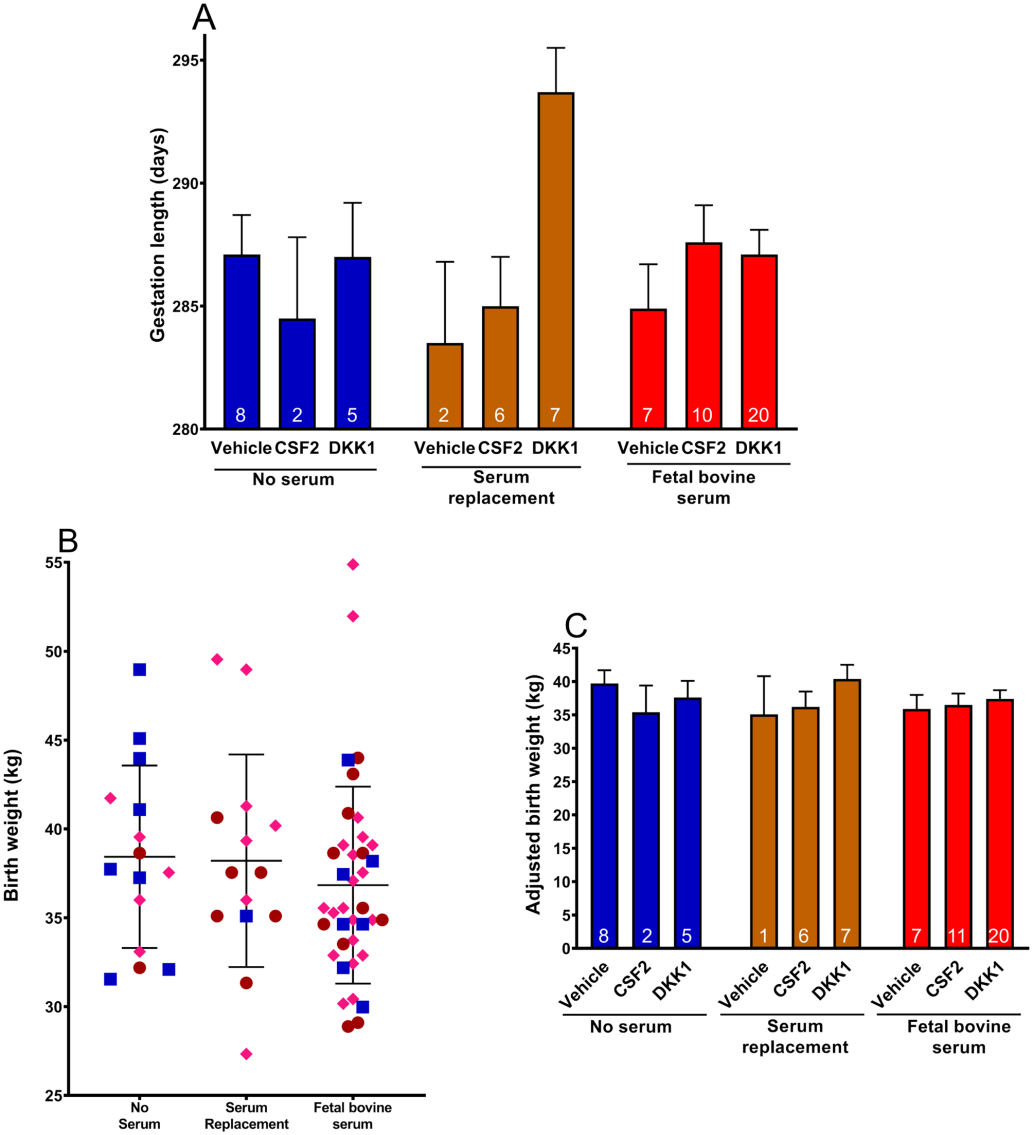
Effects of medium and embryokine treatments on gestation length and calf birth weight. Embryokine treatments were added at day 5 of culture and were vehicle, colony stimulating factor 2 (CSF2) and dickopf WNT signaling pathway inhibitor 1 (DKK1). Gestation length (**A**) was affected by embryokine (P = 0.024) and the interaction between medium and embryokine (P = 0.031). Moreover, the contrast comparing vehicle vs DKK1 across media was significant (P = 0.017). Birth weights for individual male calves after adjustment for day of age at weighing and sire are shown in panel (**B**) while results of statistical analysis are in panel (**C**). There were no treatment effects on birth weight. Results in panels (**A**,**C**) are least-squares means ± SEM.

Data on gestation length are shown on Fig. 2A. Gestation length was affected by embryokine (P = 0.024) and the interaction between medium and embryokine (P = 0.031). Moreover, the contrast comparing vehicle vs DKK1 was significant (P = 0.017). These statistical effects reflect a longer gestation length for DKK1 for the SR medium only.

### Calf birth weight

Data were available on 67 male calves from one farm (n = 23 from sire 1 and n = 44 from sire 2). Birth weights for individual male calves are presented in Fig. 2B. The three largest calves were derived from embryos produced with FBS and DKK1 (54.9 and 52.0 kg) or SR and DKK1 (49.5 kg). There were no treatment effects on birth weight (Fig. 2C). This was true regardless of whether gestation length was used as a covariate.

### Sire effects

Sire affected cleavage rate (P < 0.0001) and the percent of putative zygotes becoming blastocysts at day 6 (P = 0.052) but not the percent of cleaved embryos becoming blastocysts at day 6 or the percent of putative zygotes or cleaved embryos becoming blastocysts at day 7. Overall, sire 1 had lower cleavage rate than sire 2 (69.7 ± 2.6% vs 82.0 ± 1.5%) and lower percent of putative zygotes becoming blastocysts at day 6 (15.2 ± 1.2% vs 18.8 ± 1.5%). The percent of cleaved embryos becoming blastocysts at day 7 were 29.5 ± 2.3% for sire 1 vs 30.0 ± 1.7 for sire 2. There were sire by medium (P < 0.001) and sire by medium by embryokine interactions (P = 0.0014) for cleavage rate but treatment effects were generally in the same direction for both sires.

Embryos produced by both sires were only transferred at 5 of the 7 farms in which the experiment was conducted. An analysis of pregnancy rate at day 30 using data from the 5 farms was conducted to determine if sire affected pregnancy rate at day 30 or if there were interactions of sire with medium or embryokine. Results were available for 1259 transfers (Table 3). Pregnancy rate at day 30 was affected by sire (P < 0.0001) but not by interactions of sire with medium (P = 0.222), embryokine (P = 0.516) or medium × embryokine (P = 0.516). Pregnancy rate was higher for embryos from sire 1 than from sire 2.

**Table 3:** Effect of sire used for in vitro fertilization on pregnancy outcome at day 30 of gestation, calving rate and pregnancy loss.

Sire	Complete data set^a^	Subset of data^b^		
	Pregnant at day 30, fraction and percent	Pregnant at day 30, fraction and percent^b^	Calving rate, fraction and percent^b^	Pregnancy loss, day 30 to calving, fraction and percent^b^
1	244/486 (50.2%)	25/40 (62.5%)	23/40 (57.6%)	2/25 (8.0%)
2	264/773 (34.2%)	84/226 (37.2%)	49/226 (21.7%)	35/84 (41.7%)
P	< 0.0001	0.008	< 0.001	0.017

^a^Data from 5 farms in which embryos produced from both sires were used.

^b^Data from 1 farm in which calving information was recorded.

The farm that provided calving data included calves born by sire 1 (n = 23) and 2 (n = 44). Pregnancy rate at day 30 (P = 0.008), calving rate (P < 0.001) and pregnancy loss between day 30 and calving (P = 0.017) was affected by sire, with pregnancy rate at day 30 and calving rate being higher for sire 1 and pregnancy loss between day 30 and calving being lower for sire 1. Gestation length was affected by sire (P = 0.0008) but not by any interaction with sire. Gestation length was shorter (P = 0.001) for calves from sire 1 (283.7 ± 1.2 days vs 289.6 ± 0.9 days) but there were no interactions of other treatments with sire. There were also no effects of sire or interactions with sire on birth weight of male calves. Birth weights were 36.9 ± 1.5 kg for sire 1 and 37.2 ± 1.1 kg for sire 2.

## Discussion

This experiment was conducted to explore use of FBS and a commercial serum replacement (KnockOut Serum Replacement) and selected embryokines (CSF2 and DKK1) to increase blastocyst yield and pregnancy outcomes using in vitro production of bovine embryos. One important finding of the study was that treatments that increase blastocyst production do not necessarily improve competence of the blastocyst to establish pregnancy after transfer. Both SR and FBS caused a large increase in development of embryos to the blastocyst stage but pregnancy rates after transfer were reduced by both SR and FBS. Another important finding was that actions of embryokines depend upon the specific conditions associated with embryo production. Neither CSF2 nor DKK1 increased development to the blastocyst stage. Moreover, effects on pregnancy rate depended upon culture medium. Both molecules were detrimental to pregnancy after transfer when there was no serum in the medium but had no negative effect in the presence of SR or FBS. Rather, CSF2 increased pregnancy rate at day 30 when embryos were cultured in FBS and CSF2 and DKK1 increased calving rate when embryos were cultured in SR or FBS. The third important finding was that the sire used to produce embryos can have a large effect on establishment and maintenance of gestation, as evidenced by the large differences in pregnancy and calving rates and fetal losses between embryos derived from sires 1 and 2.

Fetal bovine serum is frequently added to embryo culture medium under commercial conditions because, as shown here and elsewhere^6,9,10^, it can increase the proportion of cultured embryos that become blastocysts. KnockOut serum replacement also caused an improvement in development to the blastocyst stage, similar to what was reported earlier^9^. Despite the improvement in development to the blastocyst stage, embryos produced in either SR or FBS were less likely to result in a pregnancy after transfer to recipient. This was especially true for SR when considering development to term. Thompson et al.^6^ also noticed that pregnancy rates were lower for embryos produced in serum as compared to embryos produced in serum-free medium. Kuran et al.^37^ reported that blastocysts produced in serum had reduced diameter, cell number and protein synthetic rate than embryos produced in absence of serum. One possibility is that there are constituents in serum that have deleterious effects on development. Alternatively, SR or FBS do not cause negative effects on development per se but more blastocysts from these treatments are derived from defective embryos that were rescued to become blastocysts. Consistent with this latter idea was the observation that negative effects of FBS on pregnancy rate were reversed if CSF2 was present in the medium and effects of SR and FBS on calving rate were reversed if CSF2 or DKK1 was present in the medium. In particular, CSF2 can exert protective effects on the embryo exposed to stresses like heat shock^27^ and cryopreservation^38^. Many of the genes whose expression in the blastocyst are altered by CSF2 are also involved in stress responses^39^. Less is known about the biology of DKK1 but effects on trophectoderm development^21^ and trophoblast elongation^24^ might rescue some embryos that might otherwise fail to develop after transfer.

Treatment with serum can also lead to large offspring syndrome in sheep^13,14^. The large offspring syndrome also occurs in cattle^40^ but there is no experimental evidence that it is increased in frequency by the presence of serum in the medium. There was no significant effect of treatment on birth weight. A similar lack of effect of serum on birth weight was reported by Estrada-Cortés et al.^10^. The three largest calves were derived from embryos treated with SR or FBS; all were also exposed to DKK1. Larger numbers are required to determine whether these treatments are associated with increased frequency of large calves.

There was no effect of CSF2 on development of embryos to the blastocyst stage. In other studies, actions of CSF2 on development have been inconsistent with CSF2 either increasing the percent becoming blastocysts^10,20,25^ or having no effect^39^. One of the determinants of the actions of CSF2 are sex, with greater effects for female embryos than male embryos^41^. Moreover, a large study by Dobbs et al.^26^ was indicative that CSF2 increased percent blastocyst when the overall amount of blastocyst development was low, had no effect when the overall development was intermediate, and had negative effects when overall development was high.

The negative effects of CSF2 on competence of embryos produced in the absence of serum to survive after transfer are in contradiction to results of earlier experiments. Specifically, CSF2 increased competence of blastocysts to establish pregnancy after transfer in two of three studies with Holstein embryos produced with X-sorted semen^20,21^. There was no effect significant effect of CSF2 on pregnancy rate of Brahman embryos produced in the absence of serum when transferred into beef recipients although pregnancy rate was numerically lower for the CSF2 group^10^. There was also no effect of CSF2 on pregnancy rate of embryos produced in serum-containing medium in beef cattle^30^. Taken together, effects of CSF2 on competence of the blastocyst to establish pregnancy have been inconsistent and it is not possible to predict conditions in which the embryokine repeatedly improves embryo competence to establish pregnancy.

Treatment of embryos with DKK1 was not expected to increase the percent that reached the blastocyst stage^21,30^ but there is one report that DKK1 treatment increased pregnancy rate when Holstein embryos produced with X-sorted semen in a serum-free medium were transferred into heat-stressed Holstein recipients^21^. In contrast, there was no effect of DKK1 on pregnancy outcomes after transfer of embryos produced in serum-containing medium into beef recipients^30^. These previous results are different than those found in the current experiment—a negative effect of DKK1 on pregnancy rate in serum-free medium and a positive effect on calving rate when SR or FBS was added to the medium. Further experiments are required to determine whether actions of DKK1 on pregnancy establishment depend upon blastocyst breed or sex or type of recipient.

An unresolved question is whether embryokines can have synergistic or antagonistic actions on the embryo when added together to culture medium. Limitations in the number of recipients precluded evaluation of whether embryos treated with both CSF2 and DKK1 differed from embryos treated with one embryokine only. In an earlier small-scale study, there was no effect of the combination of CSF2 and DKK1 to serum-containing medium on any characteristics of embryo production or outcomes of embryo transfer^30^. It is likely that optimal development of embryos in culture will require exposure of the embryo to a cocktail of embryokines which together affect several aspects of embryo development and physiology. There are a large number of genes encoding for cell-signaling receptors expressed by the bovine embryo^42^. There has been one experiment evaluating a combination of embryokines (epidermal growth factor, insulin-like growth factor 1 and fibroblast growth factor 2) on pregnancy rates after transfer of in vitro produced embryos^43^ but results were inconclusive. More work on identification of the optimal mix of embryokines is warranted.

It is well established that the specific bull used to produce an embryo can influence the proportion of embryos that develop to the blastocyst stage in culture^31–34^. Such a phenomenon was observed in the present experiment, where cleaved embryos produced using semen from sire 1 being less likely to become blastocysts than embryos from sire 2. Effects of sire on development could represent inheritance of paternal alleles by the embryo that are detrimental to embryonic development^32,33,44^ or environmental effects during spermatogenesis^45,46^ or after ejaculation^47,48^ that affect sperm function.

There were also large sire effects on development after the blastocyst stage, as evidenced by significant differences in pregnancy rate at day 30, calving rate and pregnancy loss between day 30 and term. In this case, embryos produced by sire 1 were more likely to establish and maintain pregnancy than embryos produced by sire 2. Thus, examination of sire differences at the blastocyst stage was not predictive of sire differences in subsequent embryonic survival. There is one report that sire affects pregnancy success after embryo transfer^35^ as well as a report that sire affects pregnancy loss after day 30 of gestation in inseminated cattle^49^. One implication of the large sire effects on embryonic function seen here is that screening of sires for embryo competence to develop to term could improve embryo transfer outcomes. Perhaps, there are molecular signatures of paternal contributions to embryo competence that could be used to identify superior sires. More broadly, understanding the genetic and environmental bases for variation among sires in paternal contribution to embryo competence for development to term could result in methods for improving fertility.

Several conclusions can be derived from this work. Addition of SR or FBS into embryo culture medium can improve blastocyst yield in an in vitro production system but the resultant embryos have reduced competence to establish pregnancy after transfer. Effects of CSF2 and DKK1 on embryo competence for development to term was negative in the absence of SR or FBS but positive when either SR or FBS was added to culture medium. More research is needed to understand conditions in which addition of embryokines to culture media improve pregnancy outcomes. Finally, results demonstrate the importance of sire for determining not only development of embryos to the blastocyst stage but also on establishment and maintenance of pregnancy after transfer to recipients.

## Supplementary Information

Supplementary Information.

## Data Availability

Original data are available at mendelay.com (https://doi.org/10.17632/6np5d6ts53.1).
