# Supplementary material for: Actions of CSF2 and DKK1 on bovine embryo development and pregnancy outcomes are affected by composition of embryo culture medium
Source: Sci Rep. 2022 May 7;12:7503. doi: 10.1038/s41598-022-11447-7 (PMC9079070; doi:10.1038/s41598-022-11447-7)
Supplement: Supplementary file 1 — Supplementary Information. [file 41598_2022_11447_MOESM1_ESM.pdf]

Supplementary Table S1. Formulation of embryo culture medium.

Ingredient	Amount
Water for embryo transfer – suitable for mouse embryo	1000 ml
Myo-inositol	0.5 g
$Na_3C_6H_5O_7$	0.1 g
NaCl	6.29 g
KCl	0.534 g
KH_2PO_4	0.162 g
$MgSO_4 \cdot 7H_2O$	0.182 g
Sodium lactate 60%	600 µl
$NaHCO_3$	2.1 g
$C_3H_3NaO_3$	0.08 g
$CaCl_2 \cdot 2H_2O$	0.262 g
BME Amino Acids Solution (50x)	30 ml
MEM Non-essential Amino Acid Solution (100x)	10 ml
GlutaMax™ supplement	1000 µl
